# Body mass index-dependent immunological profile changes after left ventricular assist device implantation

**DOI:** 10.3389/fimmu.2023.1256725

**Published:** 2023-10-10

**Authors:** Kristin Klaeske, Eva Katharina Messer, Sara Klein, Franz Sieg, Sandra Eifert, Josephina Haunschild, Khalil Jawad, Diyar Saeed, Alexey Dashkevich, Michael A. Borger, Maja-Theresa Dieterlen

**Affiliations:** University Clinic of Cardiac Surgery, Leipzig Heart Center, HELIOS Clinic, Leipzig, Germany

**Keywords:** body mass index, obesity, regulatory T cells, dendritic cells, immune system, left ventricular assist device

## Abstract

**Purpose:**

Infection is a common complication following left ventricular assist device (LVAD) implantation. Patients with obesity are particularly at risk due to their high percentage of adipose tissue and the resulting chronic inflammatory state and resulting immunological changes. This study investigated changes of immunological parameters in relation to body mass index (BMI) during the first year after LVAD implantation.

**Methods:**

Blood samples were obtained prior to LVAD implantation and at 3 (1^st^ FU), 6 (2^nd^ FU) and 12 mo (3^rd^ FU) after LVAD implantation. Patients were divided into three groups (normal weight: BMI of 18.5-24.9 kg/m^2^; n=12; pre-obesity: 25.0-29.9 kg/m^2^; n=15; obesity: ≥ 30.0 kg/m^2^; n=17) based on their BMI at the time of LVAD implantation. Flow cytometric analyses for CD4^+^ and CD8^+^ T cells, regulatory T cells (T_regs_), B cells as well as dendritic cells (DCs) were performed.

**Results:**

After LVAD implantation, obese patients (0.51 ± 0.20%) showed a higher proportion of overall DCs than normal-weight (0.28 ± 0.10%) and pre-obese patients (0.32 ± 0.11%, p<0.01) at 3^rd^ FU. The proportion of BDCA3^+^ myeloid DCs was lower in obese patients (64.3 ± 26.5%) compared to normal-weight patients (82.7 ± 10.0%, p_normal-weight vs. obesity_=0.05) at 2^nd^ FU after LVAD implantation. The analysis of BDCA4^+^ plasmacytoid DCs revealed a reduced proportion in pre-obese (21.1 ± 9.8%, p_normal-weight vs. pre-obesity_=0.01) and obese patients (23.7 ± 10.6%, p_normal-weight vs. obesity_=0.05) compared to normal-weight patients (33.1 ± 8.2%) in the 1^st^ FU. T cell analysis showed that CD4^+^ T cells of obese patients (62.4 ± 9.0%) significantly increased in comparison to pre-obese patients (52.7 ± 10.0%, p_pre-obesity vs. obesity_=0.05) and CD8^+^ T cells were lower in obese patients (31.8 ± 8.5%) than in normal-weight patients (42.4 ± 14.2%; p_normal-weight vs. obesity_=0.04) at the 3^rd^ FU. Furthermore, we observed significantly reduced proportions of T_regs_ in pre-obese patients compared to normal-weight and obese patients at 2^nd^ FU (p=0.02) and 3^rd^ FU (p=0.01) after LVAD implantation.

**Conclusion:**

This study reported changes of the innate and adaptive immune system of pre-obese and obese compared to normal-weight patients one year after LVAD implantation. DCs and their subsets, CD8^+^ T cells and T_regs_ were affected immune cell populations that indicate immunological changes which might increase the incidence of postoperative infection.

## 1 Introduction

The implantation of a left ventricular assist device (LVAD) has become an important therapeutic option in the treatment of end-stage heart failure (HF). Infections remain the most common complication in patients with LVAD, with an incidence ranging from 20% to 60%. The rate of infection is highest in the first year after LVAD implantation with up to 71% for driveline infections and 27% for bloodstream infection (1). Later, the infection rate is 6% per patient’s year. Serious infections contribute to higher morbidity and mortality in LVAD patients (2, 3).

Previous studies have identified predisposing factors for infections after LVAD implantation. The pre-existing inflammatory profile of end-stage HF patients combined with high levels of shear stress and foreign materials of the LVAD pump may affect the cellular immunity and thereby the inflammatory response to infection after LVAD implantation (2, 4). In this context, increased apoptotic activity in CD4^+^ T cells, impaired function of T cells due to elevated suppressive regulatory T cells (T_regs_) as well as cytokine imbalance was observed immediately after LVAD implantation (5, 6). Additionally, deactivation of monocytes was documented in LVAD patients, and could be associated with mortality in the early postoperative period (7). The reported dysfunction of both innateand adaptive immune cells may contribute to an increased risk for life-threatening infection in LVAD patients.

Obesity has been associated with an increased risk for infection in patients with LVAD support (8–10). Especially obese patients with an elevated body mass index (BMI) ≥ 30 suffer from immunological alterations and an increased risk for major infectious complications (11). This is caused by the chronic, low-grade inflammation status in the adipose tissue. Several studies have demonstrated the immunomodulatory effect of pro-inflammatory cytokines secreted by adipose tissue on various immune cells (12). Obesity may affect the proliferation and function of T cells (13–15), the number and functional properties of T_regs_ (13, 16), B cell function (17) and the immune activation through the interaction with dendritic cells (DCs) (18). Thus, it can be concluded that immunological differences exist between normal-weight, pre-obese and obese LVAD patients.

In the present study, we investigated subsets of DCs as part of the innate immune system and cell subsets of the adaptive immune system including CD3^+^, CD4^+^ and CD8^+^ T cells, T_regs_ and CD19^+^ B cells in the first year of LVAD support to identify the immunological differences of pre-obese and obese patients after LVAD implantation compared to normal-weight patients.

## 2 Materials and methods

### 2.1 Study groups and clinical characteristics

The present study was approved by the Ethics Committee of the Medical Faculty, University of Leipzig, Germany (ID:225/17-ek), and was performed according to the guidelines of the Declaration of Helsinki (2013). All patients gave their written informed consent before study initiation. In total, we studied 44 HF patients undergoing LVAD implantation between September 2018 and January 2021 at the Heart Center Leipzig, Germany. Study patients were divided into three groups based on BMI classification according to WHO criteria at the time of LVAD implantation: normal-weight patients (BMI 18.5-24.9 kg/m^2^, n = 12), pre-obese patients (BMI 25.0-29.9 kg/m^2^, n = 15), and obese patients (BMI ≥ 30 kg/m^2^, n = 17). Demographic and clinical data were collected. The postoperative course of the occurrence and type of infection was documented. Infection was defined according to the definition of the International Society for Heart and Lung Transplantation (ISHLT) and divided into 3 types: LVAD-specific, LVAD-related and non-LVAD infection (19). LVAD-specific infections are related to the device, do not occur in non-LVAD patients, and comprise pump, cannula, pocket or driveline infections. LVAD-related infections can be associated with the implanted device and include for example infective endocarditis, LVAD-related bloodstream infection, mediastinitis or wound infection. Non-LVAD infection comprise infections that are not affected by the LVAD such as respiratory tract infection, urinary tract infection, and Clostridium difficile infection (19).

### 2.2 Blood sampling

Citrated whole blood and serum were collected prior to LVAD implantation and during the follow-up (FU) period at 3 mo (1^st^ FU), 6 mo (2^nd^ FU) and 12 mo (3^rd^ FU) after LVAD implantation. Citrated blood samples were used for flow cytometric analyses of immunological cell populations. Sera were centrifuged at 2000 x g for 10 min, aliquoted and stored at -20°C until cytokine quantification.

### 2.3 Blood count analysis

Blood count analysis was conducted by the MVZ Laboratory Dr. Reising-Ackermann (Leipzig, Germany) and included the measurement of the hematocrit, hemoglobin, erythrocytes, leukocytes, platelets, lymphocytes, monocytes, neutrophil, eosinophil and basophil granulocytes and C-reactive protein (CRP).

### 2.4 Flow cytometry

Citrated blood samples were treated as described previously (20, 21). Total CD3^+^ T cells, CD4^+^ and CD8^+^ T cells as well as the degree of terminal differentiation/senescence (CD57) and activation (CD25) was quantified. T_regs_ were defined as CD3^+^CD4^+^ cells with high CD25 expression and low CD127 expression. B cells were defined as CD19 expressing lymphocytes. Peripheral blood DC subsets were defined as lineage cocktail-1^-^ (including antibodies against CD3, CD14, CD16, CD19, CD20 and CD56) and HLA-DR^+^ cells, and further characterized by blood dendritic cell antigen (BDCA) expression. BDCA1- and BDCA3-expressing DCs are myeloid DCs (mDCs), while BDCA2- and BDCA4-expressing DCs are plasmacytoid DCs (pDCs).

For cell subset staining, blood samples were incubated with different antibody panels for 20 min at room temperature: panel A: lineage cocktail-1-FITC, HLA-DR-PerCP, BDCA4-APC, BDCA2-PE; panel B: lineage cocktail-1-FITC, HLA-DR-PerCP, BDCA3-APC, BDCA1-PE; panel C: CD3-PerCP/Cy5.5, CD4-APC-H7, CD25-PE-Cy7, CD8-FITC, CD57-APC; panel D: CD3-PerCP/Cy5.5, CD4-APC-H7, CD25-PE-Cy7, CD127-Alexa Fluor 647; panel E: CD3-PerCP/Cy5.5, CD19-PE. The antibodies were purchased from BD Biosciences (Franklin Lakes, NJ, USA) and BioLegend (San Diego, CA, USA). Then, 2 mL of FACS lysing solution (BD Biosciences) were added to the samples followed by incubation for 10 min. After centrifugation at 300 *g for 5 min at room temperature, the cells were washed with 4 mL phosphate-buffered saline (PBS). The cells were fixed by adding 500 µL of 1% formalin-PBS. Analysis was performed using a *BD LSR II Flow Cytometer* and *BD FACSDiva version 6.1.3* software *(*both BD Biosciences). Standardization of the instrument was performed by weekly measurements of Cytometer Setup and Tracking Beads (BD Biosciences). Debris was excluded using a loose gate in a FSC-area vs. SSC-area dot plot. 100,000 events were recorded in each panel. Gating strategies were reported in more detail in **Supplementary Figures 1**–**4**.

### 2.5 Quantification of cytokines

Serum concentrations of the cytokines interleukin (IL)-1β, IL-2, IL-4, IL-6, IL-10, IL-17A and interferon (IFN)-γ were quantified using the Bio-Plex Pro Human Screening Panel 5plx EXP (Bio-Rad, Hercules, CA, USA) according to the manufacturer’s instructions. For assay analysis, a Luminex® 200 device and Luminex XPonent® software version 3.1 (Luminex, Austin, TX, USA) were used. Tumor necrosis factor (TNF)-α was quantified using ELISA MAX™ Deluxe Sets (BioLegend) according to the recommended protocols of the manufacturer and the Tecan reader Infinite PRO 200 and the i-control™ software (both Tecan Group AG, Männedorf, Switzerland).

### 2.6 Statistics

Data were collected and evaluated using Microsoft Excel 2016 software (Microsoft Corporation, Redmond, WA, USA). Statistical analyses were performed using SPSS Statistics 28 software (IBM Corp., New York, USA, 1989). Unless stated otherwise, data are presented as mean ± standard deviation for continuous data or as percentage proportion for categorical variables. The comparison of means for demographic and clinical parameters between the study groups was performed using the Pearson χ² test or the Yates continuity correction in case of categorical data.

Metric data were analyzed using the one-way analysis of variance (ANOVA). Levene’s test was used to test the homogeneity of variance. In case of equality of variances, the ANOVA and Scheffe *post hoc* test were used. For variance inequality, the Welch test and Dunett-T3 *post hoc* test were applied. For all analyses, p-values ≤ 0.05 were considered statistically significant.

## 3 Results

### 3.1 Clinical characteristics

In all groups, the patients were predominantly male (**Table 1**). There were no significant differences between the groups with regard to sex, etiology of cardiomyopathy, NYHA class, left ventricular ejection fraction, implant strategy, the type of LVAD device and the comorbidities arterial hypertension, diabetes mellitus type 2, chronic kidney disease, hypothyroidism, chronic inflammatory disease and chronic obstructive pulmonary disease/bronchial asthma at the time of LVAD implantation (**Table 1**). Obese patients undergoing an LVAD-implantation were younger than normal-weight patients (p_normal weight vs. obesity_ = 0.05) and developed more often hyperlipoproteinemia than normal-weight and pre-obese patients (p = 0.04). The incidence of previous implantation of a cardioverter-defibrillator or cardiac resynchronization therapy was higher in normal-weight and obese patients than in pre-obese patients (p = 0.02). Documented intolerances (p = 0.48), infectious diseases up to 6 weeks before LVAD implantation (p = 0.81), alcohol (p = 0.22) and nicotine consumption (p = 0.20) were comparable between the three groups (**Table 1**).

**Table 1:** Demographic and clinical characteristics of patients prior to LVAD implantation.

	normal weight (n = 12)	pre-obesity (n = 15)	obesity (n = 17)	p-value
age at implantation [yrs]	63.1 ± 6.9	60.0 ± 6.3	54.8 ± 11.4	0.05
male gender	11 (91.6%)	10 (66.7%)	13 (76.5%)	0.30
etiology ICM NICM	6 (50.0%) 6 (50.0%)	9 (60.0%) 6 (40.0%)	5 (29.4%) 12 (70.6%)	0.21
NYHA classification class II class III class IV	1 (8.3%) 6 (50.0%) 5 (41.7%)	0 (0%) 4 (26.7%) 11 (73.3%)	0 (0%) 10 (58.8%) 7 (41.2%)	0.17
LVEF at time of implantation 30-44% < 30%	2 (16.7%) 10 (83.3%)	1 (6.7%) 14 (93.3%)	2 (11.8%) 15 (88.2%)	0.72
indication of LVAD bridge to transplant bridge to decision destination therapy not specified	1 (8.3%) 5 (41.7%) 5 (41.7%) 1 (8.3%)	2 (13.3%) 5 (33.3%) 7 (46.7%) 1 (6.7%)	2 (11.8%) 10 (58.8%) 4 (23.5%) 1 (5.9%)	0.85
LVAD device HeartMate 3™ HVAD™	9 (75.0%) 3 (25.0%)	13 (86.7%) 2 (13.3%)	17 (100%) 0 (0%)	0.11
comorbidities arterial hypertension hyperlipoproteinemia diabetes mellitus type 2 chronic kidney disease hypothyroidism chronic inflammatory disease COPD/bronchial asthma	12 (100%) 4 (33.3%) 4 (33.3%) 6 (50.0%) 0 (0%) 0 (0%) 3 (25.0%)	15 (100%) 6 (40.0%) 8 (53.3%) 8 (53.3%) 3 (20.0%) 1 (6.7%) 0 (0%)	16 (94.1%) 13 (76.5%) 6 (35.3%) 12 (70.6%) 1 (5.9%) 1 (5.9%) 1 (5.9%)	0.44 0.04 0.48 0.46 0.17 0.67 0.07
presence of CRT/ICD	10 (83.3%)	6 (40.0%)	14 (82.4%)	0.02
prior valve surgery	3 (25.0%)	2 (13.3%)	6 (35.3%)	0.36
prior CVA	2 (16.7%)	2 (13.3%)	1 (5.9%)	0.64
prior malign disease	2 (16.7%)	2 (13.3%)	1 (5.9%)	0.64
history of chemotherapy/radiation	1 (8.3%)	1 (6.7%)	0 (0%)	0.51
infectious diseases^§^	2 (16.7%)	5 (33.3%)	5 (29.4%)	0.81
intolerances^#^	2 (16.7%)	4 (26.7%)	2 (11.8%)	0.48
history of drug abuse	0 (0%)	0 (0%)	1 (5.9%)	0.44
nicotine consumption current nicotine abuse former nicotine abuse non-smoker not specified	2 (16.7%) 7 (58.3%) 3 (25.0%) 0 (0%)	3 (20.0%) 5 (33.3%) 4 (26.7%) 3 (20.0%)	4 (23.5%) 11 (64.7%) 0 (0%) 2 (11.8%)	0.20
alcohol consumption current alcohol abuse former alcohol abuse no alcohol abuse not specified	3 (25.0%) 1 (8.3%) 7 (58.3%) 1 (8.3%)	1 (6.7%) 4 (26.7%) 8 (53.3%) 2 (13.3%)	0 (0%) 3 (17.6%) 9 (52.9%) 5 (29.4%)	0.22

^§^from 6 weeks prior to LVAD implantation, ^#^includes intolerances to medicines, foods, insect venoms. COPD, chronic obstructive pulmonary disease; CRT, cardiac resynchronisation therapy; CVA, cerebrovascular accident; HTx, heart transplantation; ICD, implantable cardioverter-defibrillator; ICM, ischaemic cardiomyopathy; LVAD, left ventricular assist device; LVEF, left ventricular ejection fraction; NICM, non-ischaemic cardiomyopathy; NYHA, New York Heart Association.

Normal-weight, pre-obese and obese patients showed comparable postoperative rates of LVAD-specific (p = 0.24), LVAD-related (p = 0.48), and non-LVAD infections (p = 0.57) (**Supplemental Table 1**). Among LVAD-specific infections, driveline infections were most frequent and affected 14 patients.

### 3.2 Blood count analysis

Prior to LVAD implantation, the blood count parameters hematocrit, hemoglobin, erythrocytes, leukocytes, platelets, lymphocytes, monocytes and granulocytes were comparable between normal-weight, pre-obese and obese patients (**Table 2**). CRP concentrations were increased due to HF but did not differ between the groups (p = 0.61).

**Table 2:** Blood count parameters before and within 12 months following LVAD implantation in normal-weight, pre-obese and obese patients.

	normal weight (n = 12)	pre-obesity (n = 15)	obesity (n = 17)	p-value
hematocrit				
pre-LVAD	0.33 ± 0.07	0.31 ± 0.06	0.34 ± 0.05	0.51
1^st^ FU	0.34 ± 0.04	0.32 ± 0.06	0.32 ± 0.06	0.52
2^nd^ FU	0.37 ± 0.06	0.33 ± 0.04	0.37 ± 0.09	0.14
3^rd^ FU	0.37 ± 0.05	0.37 ± 0.04	0.40 ± 0.06	0.27
hemoglobin [mmol/L]				
pre-LVAD	7.0 ± 1.4	6.7 ± 1.3	7.1 ± 1.2	0.62
1^st^ FU	6.9 ± 0.8	6.7 ± 1.2	6.7 ± 1.3	0.87
2^nd^ FU	7.9 ± 1.3	7.0 ± 0.8	7.3 ± 1.4	0.19
3^rd^ FU	8.0 ± 1.1	8.3 ± 1.1	8.3 ± 1.4	0.78
erythrocytes [Tpt/L]				
pre-LVAD	3.8 ± 0.8	3.7 ± 0.6	4.0 ± 0.6	0.49
1^st^ FU	3.9 ± 0.5	3.6 ± 0.7	3.9 ± 0.8	0.51
2^nd^ FU	4.3 ± 0.8	3.8 ± 0.5	4.2 ± 0.8	0.13
3^rd^ FU	4.2 ± 0.6	4.3 ± 0.6	4.7 ± 0.6	0.08
leukocytes [Gpt/L]				
pre-LVAD	7.9 ± 1.4	10.2 ± 5.0	7.5 ± 2.2	0.19
1^st^ FU	7.6 ± 2.0	8.2 ± 2.4	8.3 ± 1.8	0.70
2^nd^ FU	8.2 ± 2.5	9.0 ± 1.8	7.5 ± 2.1	0.17
3^rd^ FU	7.1 ± 1.7	10.1 ± 2.2	7.8 ± 2.2	0.01
platelets [Gpt/L]				
pre-LVAD	240 ± 89	203 ± 106	228 ± 107	0.63
1^st^ FU	346 ± 106	304 ± 92	260 ± 80	0.06
2^nd^ FU	256 ± 84	256 ± 65	215 ± 72	0.21
3^rd^ FU	222 ± 63	266 ± 44	200 ± 81	0.02
lymphocytes [Gpt/L]				
pre-LVAD	0.19 ± 0.08	0.17 ± 0.11	0.16 ± 0.09	0.75
1^st^ FU	0.20 ± 0.06	0.21 ± 0.06	0.18 ± 0.06	0.39
2^nd^ FU	0.18 ± 0.06	0.16 ± 0.07	0.17 ± 0.10	0.82
3^rd^ FU	0.20 ± 0.06	0.17 ± 0.07	0.17 ± 0.08	0.48
monocytes [%]				
pre-LVAD	0.11 ± 0.03	0.10 ± 0.04	0.10 ± 0.03	0.72
1^st^ FU	0.11 ± 0.03	0.10 ± 0.04	0.10 ± 0.02	0.70
2^nd^ FU	0.11 ± 0.04	0.10 ± 0.03	0.09 ± 0.04	0.39
3^rd^ FU	0.11 ± 0.05	0.09 ± 0.02	0.09 ± 0.03	0.35
neutrophil granulocytes [Gpt/L]				
pre-LVAD	0.68 ± 0.09	0.72 ± 0.13	0.72 ± 0.11	0.60
1^st^ FU	0.66 ± 0.05	0.65 ± 0.09	0.69 ± 0.08	0.38
2^nd^ FU	0.68 ± 0.08	0.71 ± 0.09	0.72 ± 0.13	0.59
3^rd^ FU	0.67 ± 0.09	0.71 ± 0.08	0.72 ± 0.10	0.38
eosinophil granulocytes [%]				
pre-LVAD	0.020 ± 0.016	0.011 ± 0.010	0.019 ± 0.022	0.15
1^st^ FU	0.033 ± 0.021	0.028 ± 0.010	0.026 ± 0.018	0.55
2^nd^ FU	0.028 ± 0.018	0.022 ± 0.022	0.015 ± 0.012	0.22
3^rd^ FU	0.014 ± 0.015	0.020 ± 0.014	0.016 ± 0.017	0.72
basophil granulocytes [%]				
pre-LVAD	0.009 ± 0.005	0.006 ± 0.005	0.008 ± 0.004	0.16
1^st^ FU	0.010 ± 0.004	0.012 ± 0.004	0.010 ± 0.004	0.46
2^nd^ FU	0.011 ± 0.006	0.007 ± 0.004	0.008 ± 0.004	0.16
3^rd^ FU	0.009 ± 0.003	0.009 ± 0.005	0.008 ± 0.004	0.65
CRP [mg/L]				
pre-LVAD	23.8 ± 24.4	30.3 ± 47.6	18.9 ± 17.1	0.61
1^st^ FU	5.4 ± 5.7	13.5 ± 12.8	26.5 ± 26.0	< 0.01
2^nd^ FU	3.9 ± 3.0	7.9 ± 8.9	12.9 ± 10.4	< 0.01
3^rd^ FU	2.6 ± 2.1	13.6 ± 16.9	6.1 ± 4.1	< 0.01

Measurement dates comprise the time prior to LVAD implantation (pre-LVAD), at 1^st^ FU, 2^nd^ FU and 3^rd^ FU. Reference values are: hematocrit (0.40-0.52), hemoglobin (8.7-10.9 mmol/L), erythrocytes (4.50-5.90 Tpt/L), leukocytes (4.4-11.3 Gpt/L), platelets (150-400 Gpt/L), lymphocytes (0.25-0.49 Gpt/L), monocytes (0.0-0.1%), neutrophil granulocytes (0.5-0.7 Gpt/L), eosinophil granulocytes (0.0-0.07%), basophil granulocytes (0.0-0.02%), CRP (0.0-5.0 mg/L). CRP, C-reactive protein; FU, follow-up; LVAD, left ventricular assist device; pre-LVAD, prior to LVAD implantation.

After LVAD implantation, leukocytes (3^rd^ FU: p_normal weight vs. pre-obesity_ < 0.01, p_pre-obesity vs. obesity_ = 0.02) and platelets (3^rd^ FU: p_pre-obesity vs. obesity_ = 0.03) differed between the groups, but remained within the reference range. CRP concentrations decreased in normal-weight patients, but remained at a high level in pre-obese and obese patients until the end of the first year after LVAD implantation (p_1st FU_ < 0.01, p_2ndFU_ < 0.01, p_3rdFU_ < 0.01). Significant differences of CRP levels were observed between normal-weight and obese patients (1^st^ FU: p_normal weight vs. obesity_ = 0.02; 2^nd^ FU: p_normal weight vs. obesity_ < 0.01; 3^rd^ FU p_normal weight vs. obesity_ = 0.02).

### 3.3 Flow cytometric analysis of innate immunity parameters

The flow cytometric analysis of the innate immune system comprised DC subsets (**Table 3**). In our study, the population of total DCs was comparable between the groups prior to LVAD implantation (p = 0.11), and at 1^st^ FU (p = 0.60) and 2^nd^ FU (p = 0.51) after LVAD implantation. At 3^rd^ FU after LVAD implantation, obese patients showed a higher proportion of DCs than normal-weight (p_normal-weight vs. obesity_ < 0.01) and pre-obese patients (p_normal weight vs. pre-obesity_ < 0.01).

**Table 3:** Percentages of total dendritic cells, myeloid DCs (BDCA1, BDCA3) and plasmacytoid DCs (BDCA2, BDCA4) before and within 12 months following LVAD implantation in normal-weight, pre-obese and obese patients.

	normal weight (n = 12)	pre-obesity (n = 15)	obesity (n = 17)	p-value
total DCs [% PBMCs]				
pre-LVAD	0.50 ± 0.33	0.43 ± 0.19	0.67 ± 0.39	0.11
1^st^ FU	0.43 ± 0.14	0.49 ± 0.18	0.48 ± 0.18	0.60
2^nd^ FU	0.37 ± 0.14	0.43 ± 0.20	0.44 ± 0.15	0.51
3^rd^ FU	0.28 ± 0.10	0.32 ± 0.11	0.51 ± 0.20	< 0.01
BDCA1^+^ [% total DCs]				
pre-LVAD	39.3 ± 9.0	42.9 ± 18.2	45.3 ± 12.8	0.53
1^st^ FU	43.5 ± 11.7	57.1 ± 19.4	51.4 ± 10.8	0.06
2^nd^ FU	52.6 ± 10.2	59.4 ± 10.0	50.3 ± 18.1	0.18
3^rd^ FU	58.3 ± 12.3	56.7 ± 10.1	57.1 ± 10.2	0.92
BDCA2^+^ [% total DCs]				
pre-LVAD	30.6 ± 12.4	24.5 ± 11.7	28.7 ± 11.6	0.40
1^st^ FU	33.1 ± 6.3	24.5 ± 12.5	26.0 ± 8.2	0.06
2^nd^ FU	31.6 ± 10.6	28.5 ± 8.3	27.7 ± 8.3	0.50
3^rd^ FU	34.5 ± 13.6	28.8 ± 6.3	32.8 ± 6.8	0.24
BDCA3^+^ [% total DCs]				
pre-LVAD	62.8 ± 24.5	61.2 ± 26.1	63.3 ± 24.3	0.97
1^st^ FU	72.7 ± 16.9	72.0 ± 15.6	73.0 ± 18.6	0.99
2^nd^ FU	82.7 ± 10.0	73.8 ± 13.9	64.3 ± 26.5	0.03
3^rd^ FU	82.0 ± 14.9	79.4 ± 14.8	74.1 ± 15.6	0.36
BDCA4^+^ [% total DCs]				
pre-LVAD	27.2 ± 9.6	23.9 ± 11.5	26.7 ± 11.0	0.68
1^st^ FU	33.1 ± 8.2	21.1 ± 9.8	23.7 ± 10.6	< 0.01
2^nd^ FU	30.4 ± 9.5	28.7 ± 8.0	26.4 ± 8.3	0.45
3^rd^ FU	29.6 ± 9.1	27.5 ± 8.2	29.5 ± 6.7	0.73

Measurement dates comprise the time prior to LVAD implantation (pre-LVAD), at 1^st^ FU, 2^nd^ FU and 3^rd^ FU. BDCA1/2/3/4, blood dendritic cell antigen 1/2/3/4; DCs, dendritic cells; FU, follow-up; PBMCs, peripheral blood mononuclear cells; pre-LVAD, prior to LVAD implantation.

Analysis of BDCA1- and BDCA3-expressing mDCs revealed that the proportion of BDCA1^+^ mDCs was comparable between the groups before and after LVAD implantation (pre-LVAD: p = 0.53; 1^st^ FU: p = 0.06; 2^nd^ FU: p = 0.18; 3^rd^ FU: p = 0.92). The proportion of BDCA3^+^ mDCs was lower in obese patients than in normal-weight patients at 2^nd^ FU after LVAD implantation (p_normal-weight vs. obesity_ = 0.05) (**Table 3**).

Analysis of BDCA2- and BDCA4-expressing pDCs revealed that the proportion of BDCA2^+^ pDCs was comparable before and after LVAD implantation (pre-LVAD: p = 0.40; 1^st^ FU: p = 0.06; 2^nd^ FU: p = 0.50; 3^rd^ FU: p = 0.24), while the proportion of BDCA4^+^ pDCs decreased significantly in pre-obese (p_normal-weight vs. pre-obesity_ = 0.01) and obese patients (p_normal-weight vs. obesity_ = 0.05) at 1^st^ FU after LVAD implantation compared to normal-weight patients, but recovered at 2^nd^ FU (p = 0.45) and 3^rd^ FU (p = 0.73) of LVAD support (**Table 3**).

### 3.4 Flow cytometric analysis of adaptive immunity parameters

**Figure 1:**
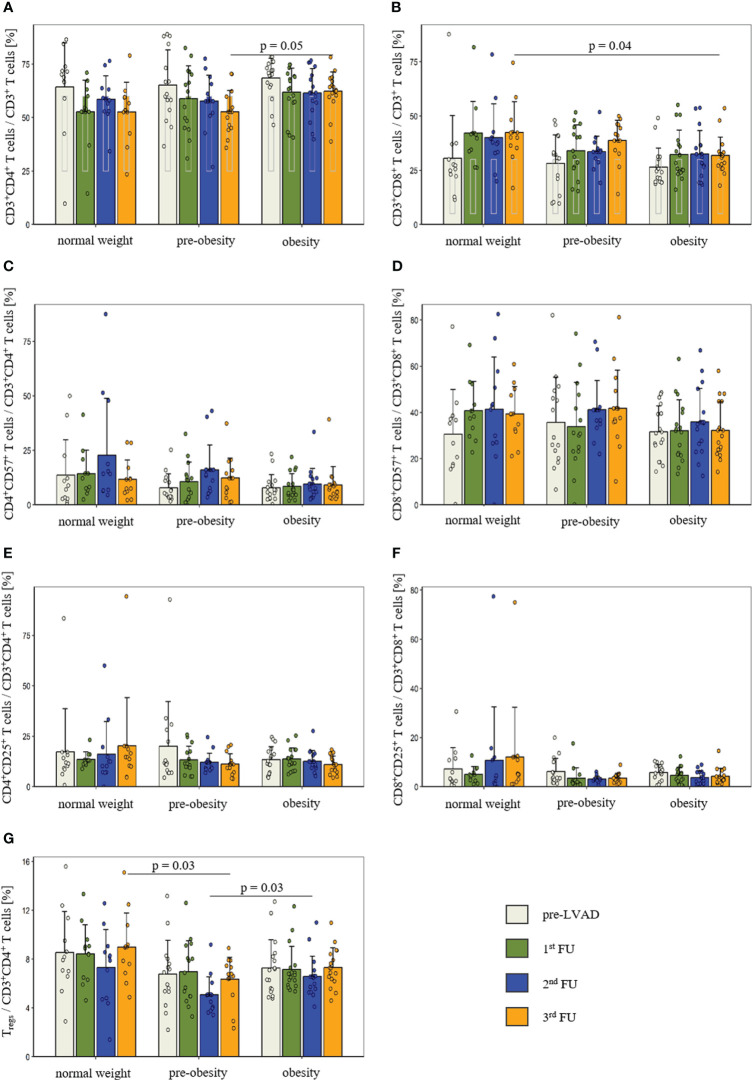
Comparison of the proportion of CD4^+^ T cells **(A)**, CD8^+^ T cells **(B)**, CD57 expression in CD4^+^ T cells **(C)** and CD8^+^ T cells **(D)**, CD25 expression in CD4^+^ T cells **(E)** and CD8^+^ T cells **(F)** as well as regulatory T cells **(G)** before and after LVAD implantation in normal-weight (n = 12), pre-obese (n = 15) and obese patients (n = 17). Measurement dates comprise the time prior to LVAD implantation (white bar), at 1^st^ FU (green bar), 2^nd^ FU (blue bar) and 3^rd^ FU (orange bar). Data are presented as mean ± standard deviation of the mean. P-values showed differences based on *post-hoc* tests. Reference values for CD4^+^ and CD8^+^ T cells are presented as grey box inside the bar. CD, cluster of differentiation; FU, follow-up; T_regs_, regulatory T cells.

The flow cytometric analysis of the adaptive immune system comprised CD3^+^ T cells, CD4^+^ and CD8^+^ T cells, T_regs_ and CD19^+^ B cells. The proportion of CD3^+^ T cells was comparable prior to LVAD implantation (p = 0.07) and at 1^st^ FU (p = 0.32), 2^nd^ FU (p = 0.09) and 3^rd^ FU (p = 0.08) after LVAD implantation. Prior to LVAD implantation, all groups showed high proportions of CD4^+^ T cells (normal weight: 64.4 ± 20.6%; pre-obesity: 65.2 ± 16.5%; obesity: 68.5 ± 9.2%, p = 0.74). The reference range for CD4^+^ T cells is 25-60% (22). At the 3^rd^ FU after LVAD implantation, CD4^+^ T cells of obese patients (62.4 ± 9.0%) remained above this range and significantly differed in comparison to pre-obese patients (52.7 ± 10.0%, p_pre-obesity vs. obesity_ = 0.05) (**Figure 1A**). Following LVAD implantation, the proportion of CD8^+^ T cells exceeded the reference range of 5-30% (22) in all groups. At the 3^rd^ FU after LVAD implantation, obese patients (31.8 ± 8.5%) had a significantly lower proportion of CD8^+^ T cells than normal-weight patients (42.4 ± 14.2%; p_normal-weight vs. obesity_ = 0.04) (**Figure 1B**).

Terminal differentiation of T cells was determined by the expression of CD57. Before and after LVAD implantation, the groups were comparable for CD57^+^ expression in CD4^+^ T cells (pre-LVAD: p = 0.50; 1^st^ FU: p = 0.19; 2^nd^ FU: p = 0.08; 3^rd^ FU: p = 0.57) and CD8^+^ T cells (pre-LVAD: p = 0.69; 1^st^ FU: p = 0.32; 2^nd^ FU: p = 0.59; 3^rd^ FU: p = 0.14) (**Figures 1C, D**).

In addition, T cell activation was examined by measuring CD25 expression. There were no detectable differences in T cell activation between the groups at any time point in CD4^+^ T cells (pre-LVAD: p = 0.57; 1^st^ FU: p = 0.97; 2^nd^ FU: p = 0.70; 3^rd^ FU: p = 0.13) and CD8^+^ T cells (pre-LVAD: p = 0.86; 1^st^ FU: p = 0.50; 2^nd^ FU: p = 0.35; 3^rd^ FU: p = 0.30) (**Figures 1E, F**).

Further, the subpopulation of CD4^+^CD25^high^CD127^low^ T_regs_ was quantified. In comparison to normal-weight and obese patients, pre-obese patients showed a reduced percentage of T_regs_ at 2^nd^ FU (normal weight: 7.3 ± 3.1%; pre-obesity: 5.1 ± 1.5%; obesity: 6.6 ± 1.7%; p = 0.02; p_pre-obesity vs. obesity_ = 0.03) and at 3^rd^ FU (normal weight: 9.0 ± 2.8%; pre-obesity: 6.4 ± 1.8%; obesity: 7.3 ± 1.6%; p = 0.01; p_normal weight vs. pre-obesity_ = 0.03) after LVAD implantation (**Figure 1G**).

The flow cytometric analysis of CD19^+^ B cells revealed no significant differences between the three groups before LVAD implantation (normal weight: 5.5 ± 2.7%; pre-obesity: 7.6 ± 5.4%; obesity: 7.3 ± 5.0%; p = 0.47), at 1^st^ FU (normal weight: 4.1 ± 1.8%; pre-obesity: 5.7 ± 2.7%; obesity: 5.4 ± 3.0%; p = 0.26), 2^nd^ FU (normal weight: 4.3 ± 2.0%; pre-obesity: 5.5 ± 2.3%; obesity: 5.8 ± 3.6%; p = 0.34) and 3^rd^ FU after LVAD implantation (normal weight: 4.6 ± 2.0%; pre-obesity: 7.0 ± 3.1%; obesity: 6.7 ± 3.4%; p = 0.08).

### 3.5 Cytokine measurement

Prior to LVAD implantation, the serum levels of the proinflammatory cytokines IL-1β (p = 0.14), IL-2 (p = 0.87), IL-6 (p = 0.65), IL-17A (p = 0.57), IFN-γ (p = 0.63) and TNF-α (p = 0.06) as well as the anti-inflammatory cytokines IL-4 (p = 0.19) and IL-10 (p = 0.23) were comparable between the three groups (**Supplementary Table 2**). During the 1^st^, 2^nd^ and 3^rd^ FU following LVAD implantation, there were no detectable differences of serum cytokine concentrations of IL-1β, IL-2, IL-6, IL-17A, IFN-γ and TNF-α as well as the anti-inflammatory cytokines IL-4 and IL-10.

## 4 Discussion

This study demonstrated the impact of obesity and pre-obesity on the immunological profile of patients undergoing LVAD implantation in the first postoperative year. Obese and pre-obese LVAD patients revealed changes affecting the innate and adaptive immune system. Regarding the innate immune system, obese patients showed higher proportions of DCs at the 3^rd^ FU after LVAD implantation compared to pre-obese and normal-weight patients. DC subset analysis revealed a reduction of BDCA3^+^ mDCs in obese patients at 2^nd^ FU and of BDCA4^+^ pDCs in pre-obese and obese patients in the 1^st^ FU after LVAD implantation. The analysis of T cells as part of the adaptive immune system showed a higher proportion of CD4^+^ T cells and a lower proportion of CD8^+^ T cells at the 3^rd^ FU in obese patients. Further, significantly reduced proportions of T_regs_ in pre-obese patients at 2^nd^ and 3^rd^ FU after LVAD implantation were detected. Although, pre-obese and obese LVAD-patients solely show temporary and slight immunological changes, the altered immune status might increase the incidence of postoperative infection in relation to BMI.

DCs are antigen-presenting innate immune cells playing an important role in activating and regulating naïve T cells and thereby modulating the immune response in obesity-induced inflammation (18). The characterization of DCs is challenging due to their low numbers in circulation, which ranges between 0.55% and 1.63% of mononuclear blood cells (23, 24). In this study, low levels of circulating DCs could be explained by the migration of cells into the myocardium caused by the progressive remodeling due to HF in these patients (25). LVAD support and ventricular alleviation did not lead to increased percentages of DCs, but higher proportions of total DCs were documented for obese patients. This increase of DCs in obese patients seems to be an LVAD-specific effect, because circulating DCs have been reported to be decreased in obesity (26). It might be speculated that LVAD support triggers DCs development in obese patients to increase the activability of the immune system. However, this is in contrast to the finding of lower proportions of BDCA3^+^ mDCs in obese patients. mDCs trigger the initial immune responses to infectious diseases (27). Thus, a decrease in mDCs might indicate a lower responsibility to infection in obese LVAD patients. In non-LVAD, obese patients, increased proportions has been found for BDCA1^+^ mDCs but not for BDCA3^+^ mDCs (28). Thus, the changes in the mDC subset seem to be LVAD-specific as well and not only triggered by obesity.

In obesity, circulating pDCs are recruited from the blood to visceral adipose tissue to maintain IFN-induced inflammation (29). This is consistent with our findings of a lower proportion of blood circulating BDCA4^+^ in pre-obese and obese LVAD patients. In addition, pDCs are able to induce T_regs_ (30). Thus, we could hypothesize that a reduction of BDCA4^+^ pDCs in pre-obese and obese patients could lead to a lower proportion of T_regs_ in these patients. The absolute numbers of T_regs_ in obese patients compared to normal-weight LVAD patients supported this hypothesis, but did not reach statistical significance, except for pre-obese patients at 2^nd^ and 3^rd^ FU after LVAD implantation. Other immune cells of the innate immune system such as monocytes, eosinophils, neutrophils and basophils are not affected from BMI-dependent immunological changes in this study cohort.

The adaptive immune response is conducted by lymphocytes including T and B cells (31). Neither the blood leukocyte concentration nor the proportion of B cells differed between normal-weight, pre-obese and obese patients in our study. The previously HF-induced alterations in B cell function could mask a BMI-related difference of these cell populations, which might explain the comparability for B cells between the study groups (32).

In obese LVAD patients, the proportion of CD4^+^ T cells was increased and proportion of CD8^+^ T cells was decreased at the 3^rd^ FU. Previous studies investigating the immune system in obese, non-LVAD patients reported an increase of CD3^+^CD4^+^ and CD8^+^ T cells (33, 34). CD4^+^ T cells contribute to the inflammatory state in both, HF and obesity (35, 36), and promote the generation of effector CD8^+^ T cells, for example, via enhanced antigen presenting cell-mediated IL-6 and TNF-α production (37). The documented changes in CD4^+^ and CD8^+^ T cells in the present study underline the hypothesis that obese LVAD patients suffer from CD8^+^ T cell reduction which could be one reason for a reduced viral defense in this patient cohort. As reported in a study of Messer et al., viral defense as well as bacterial immune reactivity seems to be affected in obese LVAD patients and comprise changes in CD8^+^ T cells and natural killer cells (21).

The expression of the T cell activation marker CD25 and the terminal differentiation marker CD57 in CD4^+^ and CD8^+^ T cells were comparable between the studied BMI groups. In obesity, CD4^+^ T cells were activated by adipocytes leading to T cell differentiation, expansion and cytokine production that contribute to the chronic inflammatory process in adipose tissue (38). However, previous cardiac remodelling in end-stage HF patients may lead to a higher percentage of circulating CD4^+^ T cells which does not further increase in obesity (35). Youn et al. suggested that senescent CD57^+^ T cells have a pathogenic potential in cardiovascular diseases and various chronic inflammatory responses like obesity (39). Therefore, the increased proportion of CD8^+^CD57^+^ T cells before LVAD implantation observed in this study could be related to obesity in these patients.

T_regs_ have been found to be decreased in pre-obese patients in the 2^nd^ and 3^rd^ FU. A reduction of T_regs_ in visceral adipose tissue has been reported by Park et al. (33) and seems to be characteristic in patients with increased metabolic risk (40). Thus, T_reg_ reduction in our study cohort seems to be an effect caused by obesity and not by LVAD support.

The observed immunological changes do not implicate that the immune responses to active infection of pre-obese and obese LVAD patients differs substantially from that of normal-weight patients. Instead, parts of the immune system seems to be weakened and influenced by pre-obesity and obesity. This might result in an impaired immunological control to pathogens in an early state and a higher rate of infection breakthrough in pre-obese and obese LVAD patients.

The present study has some limitations. First, the classification of study groups based on preoperative BMI, but additional parameters such as body fat or the hip-to-waist ratio could provide more detailed information for group assignment to avoid BMI miscalculation due to edema in end-stage HF patients before LVAD implantation. Second, it is important to consider that the immunological effects of HF in obese patients before LVAD implantation cannot be disconnected from BMI-associated effects in this study. Third, the influence of different implanted LVAD types with varying pump and flow characteristics on circulating immune cell populations cannot be excluded. This study included patients with HeartMate 3™ and HVAD™ support. However, the number of patients with HVAD™ support was too low for a comparison of both LVAD types. Continuous-flow axial and pulsatile pumps are not represented in this study. LVAD-induced shear stress is known to affect various blood components (41), but the effect on immune cells are currently not described. Fourth, the effect of postoperative infections within one year after LVAD implantation on immunological parameters cannot be eliminated. However, the low infection rate in this study might minimize this effect. Fifth, immunological investigations in a higher number of patients would increase the reliability of this study. Sixth, subsets of monocytes and macrophages should be investigated in future studies to increase the knowledge of the effects on the innate immune system.

In conclusion, this study reported immunological changes of the innate and adaptive immune system of pre-obese and obese compared to normal-weight LVAD patients in the first year after implantation. Subsets of mDCs, pDCs, CD4^+^, CD8^+^ T cells and T_regs_ were affected immune cell populations that indicate temporary and slight immunological changes that might increase the incidence of postoperative infection. These insights into immunological changes may be useful for future treatment and prevention of infection in patients with LVAD. In clinical use, ongoing weight monitoring and the motivation of patients for controlled weight loss through established physical activity programs and nutritional diet options during the FU is most important to reduce the risk of postoperative infections. In particular, pre-obese patients could rapidly benefit from these options to optimize their immunological profile and avoid infectious complications in the long-term.

## Data availability statement

The raw data supporting the conclusions of this article will be made available by the authors, without undue reservation.

## Ethics statement

The studies involving humans were approved by Ethics Committee of the Medical Faculty, University of Leipzig, Germany. The studies were conducted in accordance with the local legislation and institutional requirements. The participants provided their written informed consent to participate in this study.

## Author contributions

KK: Conceptualization, Data curation, Formal Analysis, Writing – original draft, Writing – review and editing. EM: Conceptualization, Data curation, Formal Analysis, Funding acquisition, Writing – original draft, Writing – review and editing. SK: Data curation, Writing – review and editing. FS: Data curation, Writing – review and editing. SE: Formal Analysis, Writing – review and editing. JH: Data curation, Writing – review and editing. KJ: Formal Analysis, Writing – review and editing. DS: Formal Analysis, Writing – review and editing. AD: Formal Analysis, Writing – review and editing. MB: Conceptualization, Supervision, Writing – review and editing. MD: Conceptualization, Formal Analysis, Supervision, Writing – original draft, Writing – review and editing.

## Supplementary material

Supplementary Figure 1 Flow cytometric gating strategy for dendritic cells and their subsets positive for BDCA 1-4. Following excluding debris using a loose gate in a FSC-area vs. SSC-area dot plot, HLA-DR (PerCP-labeled)-positive, lineage cocktail-1 (FITC-labeled)-negative cells were defined as dendritic cells. Staining against BDCA1 (CD1c, PE-labeled), BDCA2 (CD303, PE-labeled), BDCA3 (CD141, APC-labeled) or BDCA4 (CD304, APC-labeled) was performed to differentiate between the different subpopulations of dendritic cells. BDCA1/2/3/4, blood dendritic cell antigen; DCs, dendritic cells.

Supplementary Figure 2 Flow cytometric gating strategy for CD4- and CD8-positive T cells and their grade of terminal differentiation and activation. Following excluding debris using a loose gate in a FSC-area vs. SSC-area dot plot, CD3 (PerCP-Cy5.5-labeled)-positive cells were defined as T cells. CD3-positive T cells were subdivided into CD4- and CD8-positive cells by staining against CD4 (APC-H7-labeled) or CD8 (FITC-labeled). Terminal differentiation was assessed by staining CD4- and CD8-positive cells against CD57 (APC-labeled). Activation of CD4- and CD8-positive cells was quantified by staining CD25 (PE-Cy7-labeled).

Supplementary Figure 3 Flow cytometric gating strategy for regulatory T cells. Following excluding debris using a loose gate in a FSC-area vs. SSC-area dot plot, CD3 (PerCP-Cy5.5-labeled)-positive, CD4 (APC-H7-labeled)-positive cells were further gated for a high CD25 (PE-Cy7-labeled) expression and a low CD127 (Alexa 647-labeled) expression. Tregs, regulatory T cells.

Supplementary Figure 4 Flow cytometric gating strategy for CD19-positive B cells. Following excluding debris using a loose gate in a FSC-area vs. SSC-area dot plot, CD3 (PerCP-Cy5.5-labeled)-negative cells were further gated for their CD19 (PE-labeled) expression.
