# Supplementary figures and images for: Body mass index-dependent immunological profile changes after left ventricular assist device implantation
Source: Front Immunol. 2023 Oct 10;14:1256725. doi: 10.3389/fimmu.2023.1256725 (PMC10597783; doi:10.3389/fimmu.2023.1256725)

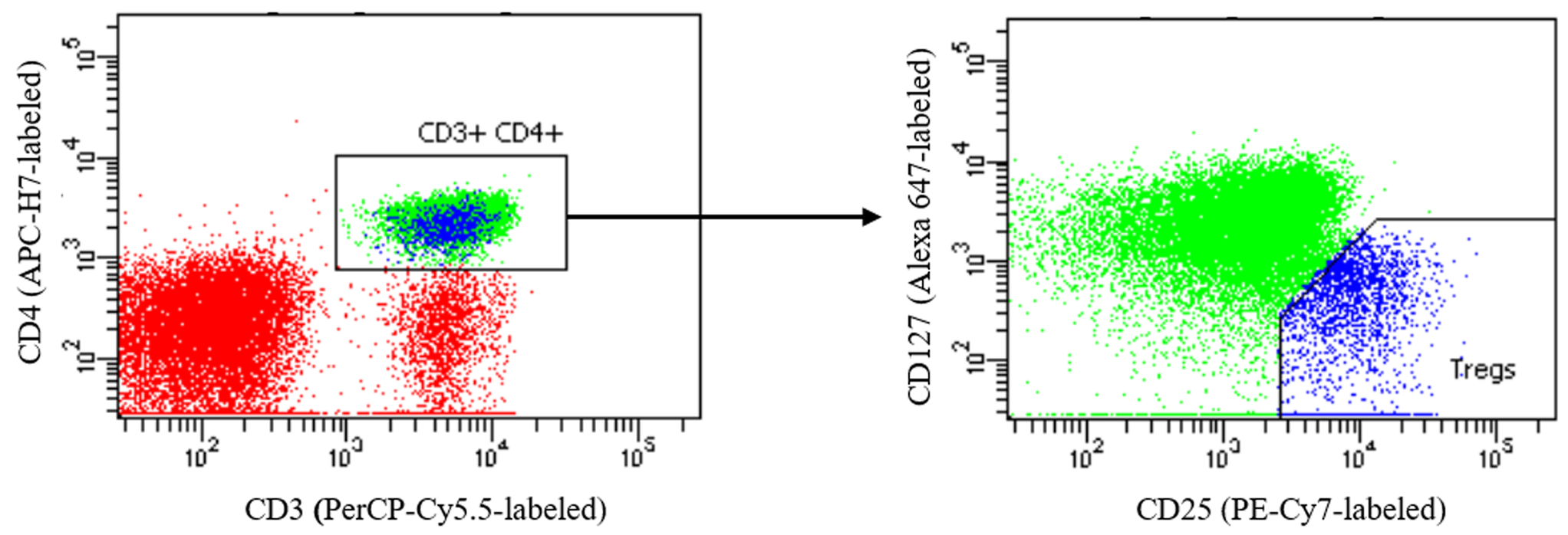

Supplement: Supplementary Figure 1 — Flow cytometric gating strategy for dendritic cells and their subsets positive for BDCA 1-4. Following excluding debris using a loose gate in a FSC-area vs. SSC-area dot plot, HLA-DR (PerCP-labeled)-positive, lineage cocktail-1 (FITC-labeled)-negative cells were defined as dendritic cells. Staining against BDCA1 (CD1c, PE-labeled), BDCA2 (CD303, PE-labeled), BDCA3 (CD141, APC-labeled) or BDCA4 (CD304, APC-labeled) was performed to differentiate between the different subpopulations of dendritic cells. BDCA1/2/3/4, blood dendritic cell antigen; DCs, dendritic cells. [file Image_1.tif]

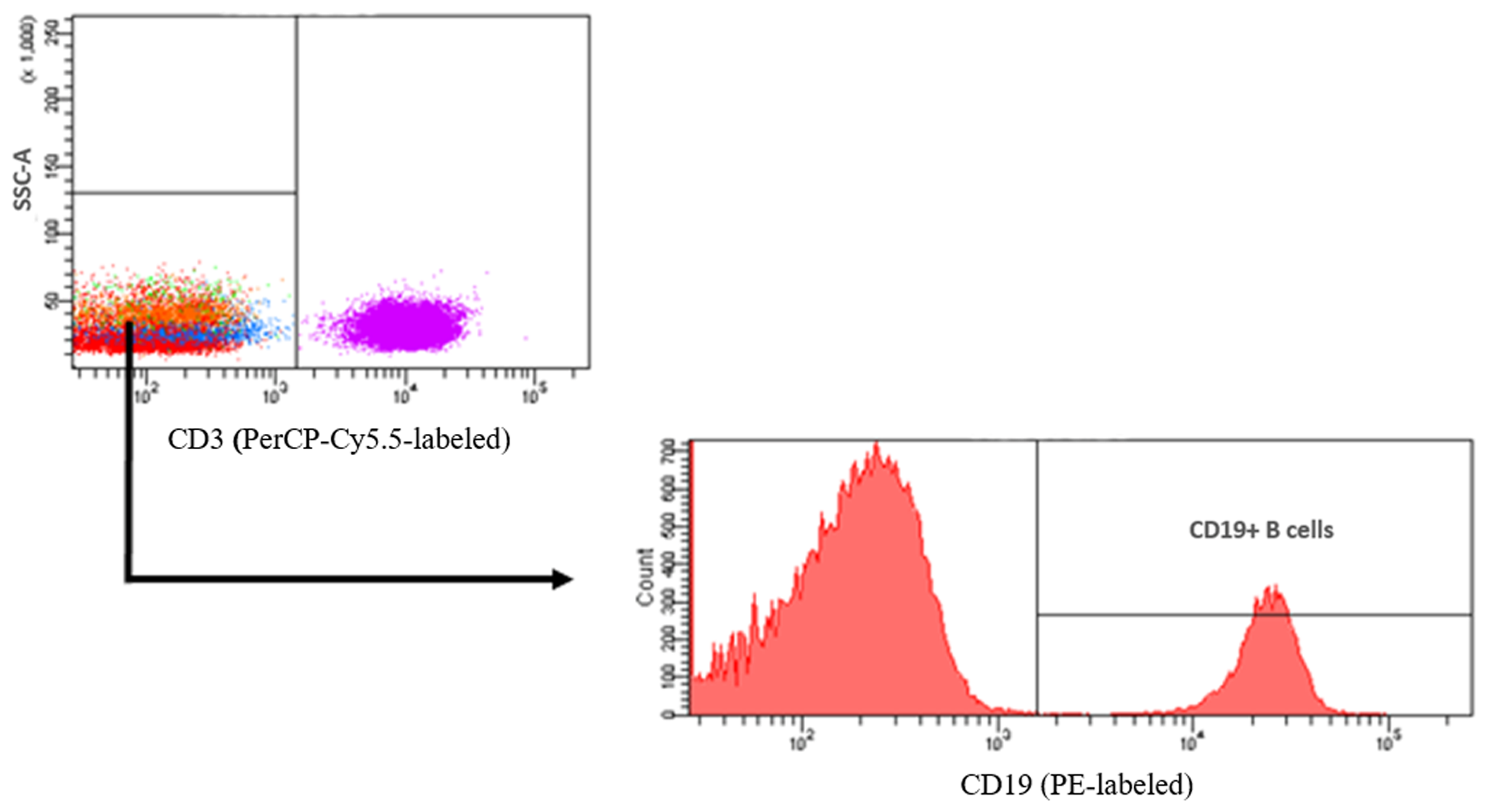

Supplement: Supplementary Figure 2 — Flow cytometric gating strategy for CD4- and CD8-positive T cells and their grade of terminal differentiation and activation. Following excluding debris using a loose gate in a FSC-area vs. SSC-area dot plot, CD3 (PerCP-Cy5.5-labeled)-positive cells were defined as T cells. CD3-positive T cells were subdivided into CD4- and CD8-positive cells by staining against CD4 (APC-H7-labeled) or CD8 (FITC-labeled). Terminal differentiation was assessed by staining CD4- and CD8-positive cells against CD57 (APC-labeled). Activation of CD4- and CD8-positive cells was quantified by staining CD25 (PE-Cy7-labeled). [file Image_2.tif]

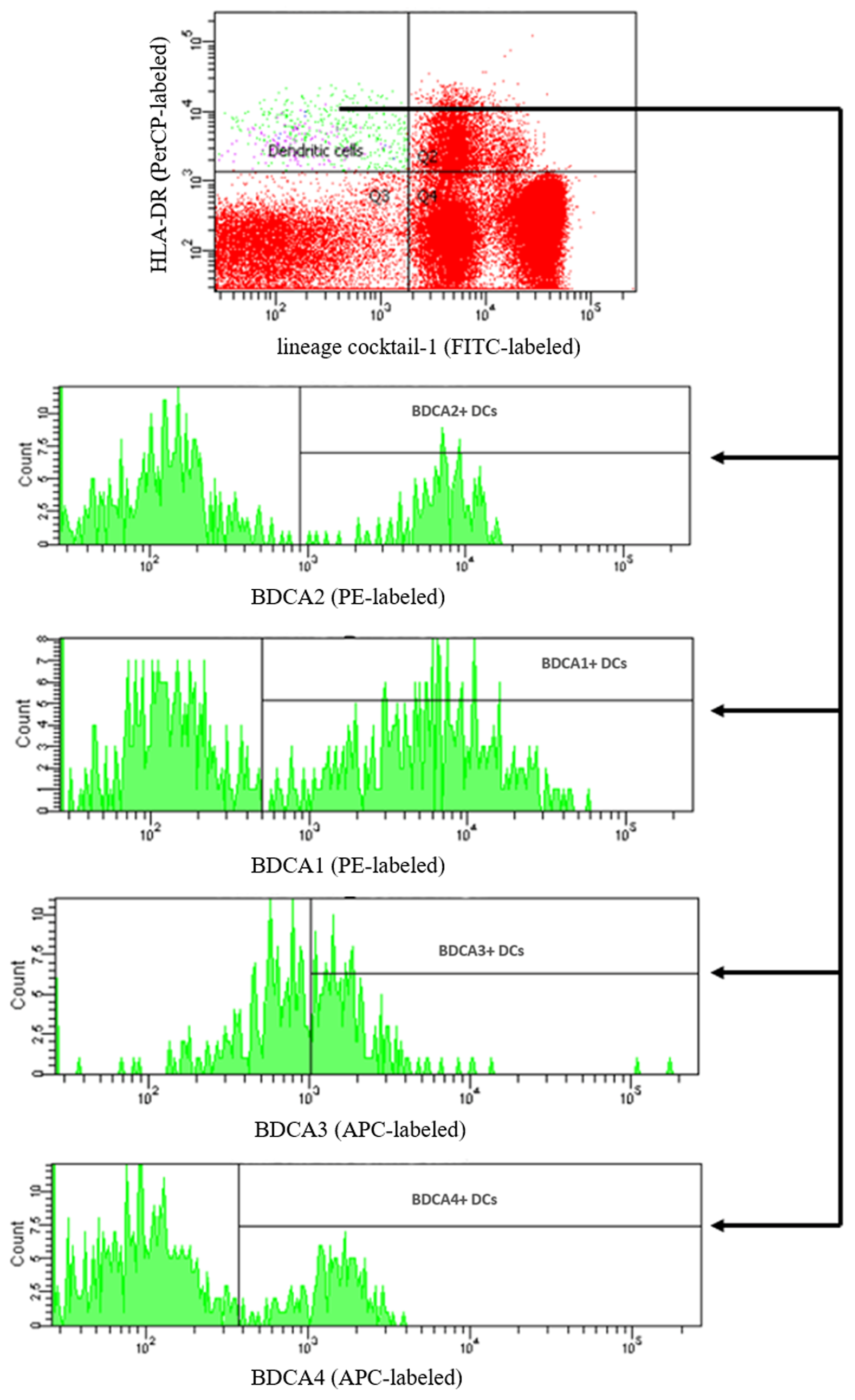

Supplement: Supplementary Figure 3 — Flow cytometric gating strategy for regulatory T cells. Following excluding debris using a loose gate in a FSC-area vs. SSC-area dot plot, CD3 (PerCP-Cy5.5-labeled)-positive, CD4 (APC-H7-labeled)-positive cells were further gated for a high CD25 (PE-Cy7-labeled) expression and a low CD127 (Alexa 647-labeled) expression. Tregs, regulatory T cells. [file Image_3.tif]

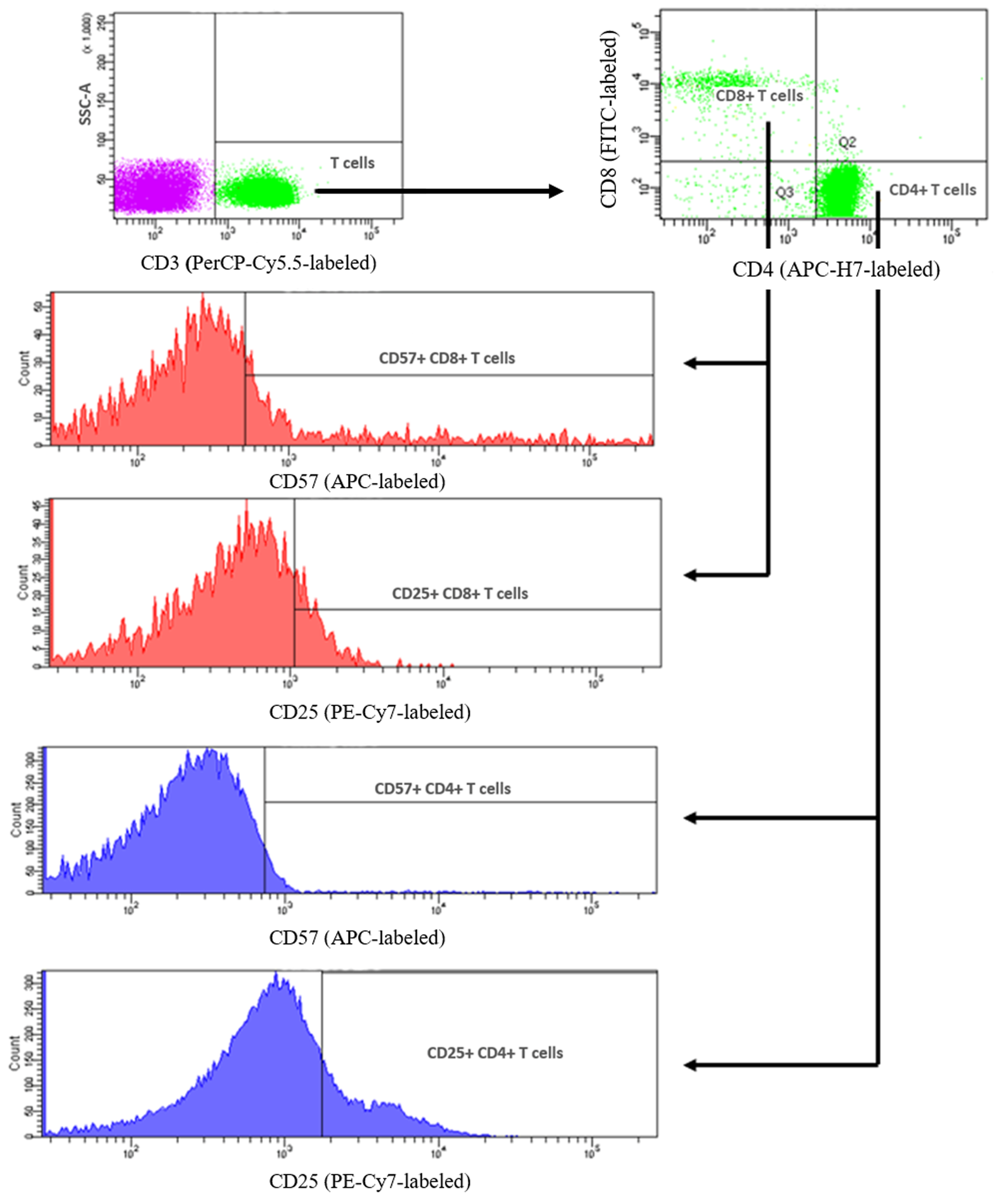

Supplement: Supplementary Figure 4 — Flow cytometric gating strategy for CD19-positive B cells. Following excluding debris using a loose gate in a FSC-area vs. SSC-area dot plot, CD3 (PerCP-Cy5.5-labeled)-negative cells were further gated for their CD19 (PE-labeled) expression. [file Image_4.tif]
