# Supplementary material for: Body mass index-dependent immunological profile changes after left ventricular assist device implantation
Source: Front Immunol. 2023 Oct 10;14:1256725. doi: 10.3389/fimmu.2023.1256725 (PMC10597783; doi:10.3389/fimmu.2023.1256725)
Supplement: Supplementary file 5 [file Table_1.docx]

**Supplementary Table 1: Occurrence of infections within 12 months following LVAD implantation in normal-weight, pre-obese and obese patients.**

|  | normal weight  (n = 12) | pre-obesity  (n = 15) | obesity  (n = 17) | p value |
| --- | --- | --- | --- | --- |
| LVAD-specific infections  percutaneous driveline infection | 2 (16.7%) | 7 (46.6%) | 5 (29.4%) | 0.24  0.24 |
| LVAD-related infections  infectious endocarditis  pathogens detected in blood culture | 1 (8.3%)  1 (8.3%) | 0 (0%)  3 (20.0%) | 1 (5.9%)  1 (5.9%) | 0.48  0.55  0.42 |
| non-LVAD infections  pulmonary infections  urinary infections  clostridium difficile infections  pathogens detected in blood culture  other | 4 (33.3%)  1 (8.3%)  0 (0%)  0 (0%)  0 (0%) | 1 (6.6%)  3 (20.0%)  0 (0%)  3 (20.0%)  1 (6.6%) | 1 (5.9%)  3 (17.6%)  2 (11.8%)  3 (17.6%)  1 (5.9%) | 0.57  0.07  0.69  0.19  0.27  0.67 |
| infections per patient | 0.9 ± 0.9 | 1.2 ± 1.4 | 1.4 ± 1.5 | 0.69 |
| time to first infection event [mo] | 0.5 ± 0.7 | 1.3 ± 2.1 | 2.2 ± 2.6 | 0.32 |

Footnote Supplementary Table 1: LVAD, left ventricular assist device; mo, months
