# Supplementary material for: Body mass index-dependent immunological profile changes after left ventricular assist device implantation
Source: Front Immunol. 2023 Oct 10;14:1256725. doi: 10.3389/fimmu.2023.1256725 (PMC10597783; doi:10.3389/fimmu.2023.1256725)
Supplement: Supplementary file 6 [file Table_2.docx]

**Supplementary Table 2: Serum levels of cytokines within 12 months following LVAD implantation in normal-weight, pre-obese and obese patients.**

|  | normal weight  (n = 12) | pre-obesity  (n = 15) | obesity  (n = 17) | p-value |
| --- | --- | --- | --- | --- |
| IFN-γ [pg/mL] |  |  |  |  |
| pre-LVAD | 0.11 $\pm$ 0.07 | 1.02 $\pm$ 0.95 | 0.09 $\pm$ 0.07 | 0.63 |
| 1^st^ FU | 0.00 $\pm$ 0.00 | 1.69 $\pm$ 1.55 | 0.00 $\pm$ 0.00 | 0.36 |
| 2^nd^ FU | 0.05 $\pm$ 0.05 | 6.70 $\pm$ 6.63 | 0.16 $\pm$ 0.10 | 0.41 |
| 3^rd^ FU | 0.00 $\pm$ 0.00 | 0.74 $\pm$ 0.74 | 0.05 $\pm$ 0.05 | 0.38 |
| IL-1β [pg/mL] |  |  |  |  |
| pre-LVAD | 0.35 $\pm$ 0.35 | 3.50 $\pm$ 1.71 | 1.12 $\pm$ 0.51 | 0.14 |
| 1^st^ FU | 1.02 $\pm$ 0.52 | 1.87 $\pm$ 0.83 | 1.40 $\pm$ 0.56 | 0.71 |
| 2^nd^ FU | 2.16 $\pm$ 1.02 | 1.54 $\pm$ 0.61 | 1.73 $\pm$ 0.80 | 0.88 |
| 3^rd^ FU | 0.60 $\pm$ 0.60 | 2.20 $\pm$ 1.42 | 2.25 $\pm$ 1.19 | 0.58 |
| IL-2 [pg/mL] |  |  |  |  |
| pre-LVAD | 0.09 $\pm$ 0.09 | 0.15 $\pm$ 0.10 | 0.09 $\pm$ 0.07 | 0.87 |
| 1^st^ FU | 0.00 $\pm$ 0.00 | 0.07 $\pm$ 0.07 | 0.20 $\pm$ 0.14 | 0.47 |
| 2^nd^ FU | 0.09 $\pm$ 0.09 | 0.00 $\pm$ 0.00 | 0.08 $\pm$0.08 | 0.68 |
| 3^rd^ FU | 0.00 $\pm$ 0.00 | 0.00 $\pm$ 0.00 | 0.00 $\pm$ 0.00 | - |
| IL-4 [pg/mL] |  |  |  |  |
| pre-LVAD | 0.00 $\pm$ 0.00 | 0.04 $\pm$ 0.03 | 0.05 $\pm$ 0.04 | 0.19 |
| 1^st^ FU | 0.00 $\pm$ 0.00 | 0.00 $\pm$ 0.00 | 0.01 $\pm$ 0.01 | 0.43 |
| 2^nd^ FU | 0.04 $\pm$ 0.03 | 0.04 $\pm$ 0.04 | 0.00 $\pm$ 0.00 | 0.51 |
| 3^rd^ FU | 0.00 $\pm$ 0.00 | 0.00 $\pm$ 0.00 | 0.03 $\pm$ 0.02 | 0.45 |
| IL-6 [pg/mL] |  |  |  |  |
| pre-LVAD | 42.0 $\pm$ 9.2 | 61.7 $\pm$ 13.3 | 58.1 $\pm$ 18.0 | 0.65 |
| 1^st^ FU | 20.5 $\pm$ 2.0 | 48.9 $\pm$ 18.7 | 43.6 $\pm$ 10.4 | 0.06 |
| 2^nd^ FU | 19.1 $\pm$ 2.0 | 33.9 $\pm$ 17.3 | 28.7 $\pm$ 4.3 | 0.14 |
| 3^rd^ FU | 15.5 $\pm$ 0.5 | 28.9 $\pm$ 7.0 | 30.5 $\pm$ 8.8 | 0.33 |
| IL-10 [pg/mL] |  |  |  |  |
| pre-LVAD | 0.00 $\pm$ 0.00 | 0.39 $\pm$ 0.32 | 0.69 $\pm$ 0.28 | 0.23 |
| 1^st^ FU | 0.00 $\pm$ 0.00 | 0.05 $\pm$ 0.05 | 0.46 $\pm$ 0.26 | 0.16 |
| 2^nd^ FU | 0.00 $\pm$ 0.00 | 0.00 $\pm$ 0.00 | 0.00 $\pm$ 0.00 | - |
| 3^rd^ FU | 0.00 $\pm$ 0.00 | 0.00 $\pm$ 0.00 | 0.54 $\pm$ 0.54 | 0.49 |
| IL-17A [pg/mL] |  |  |  |  |
| pre-LVAD | 0.31 $\pm$ 0.31 | 0.14 $\pm$ 0.12 | 0.51 $\pm$ 0.29 | 0.57 |
| 1^st^ FU | 0.00 $\pm$ 0.00 | 0.00 $\pm$ 0.00 | 0.25 $\pm$0.14 | 0.12 |
| 2^nd^ FU | 0.00 $\pm$ 0.00 | 0.00 $\pm$ 0.00 | 0.00 $\pm$ 0.00 | - |
| 3^rd^ FU | 0.07 $\pm$ 0.07 | 0.00 $\pm$ 0.00 | 0.00 $\pm$ 0.00 | 0.27 |
| TNF-α [pg/mL] |  |  |  |  |
| pre-LVAD | 0.26 $\pm$ 0.12 | 0.83 $\pm$ 0.26 | 0.81 $\pm$ 0.26 | 0.06 |
| 1^st^ FU | 0.87 $\pm$ 0.35 | 0.96 $\pm$ 0.28 | 1.42 $\pm$0.65 | 0.70 |
| 2^nd^ FU | 0.52 $\pm$ 0.20 | 0.56 $\pm$ 0.27 | 1.12 $\pm$ 0.46 | 0.40 |
| 3^rd^ FU | 0.32 $\pm$ 0.21 | 1.08 $\pm$ 0.38 | 0.79 $\pm$ 0.42 | 0.40 |

Footnote Supplementary Table 2: Measurement dates comprise the time prior to LVAD implantation (pre-LVAD), at 1^st^ FU, 2^nd^ FU and 3^rd^ FU. Data were displayed as mean $\pm$ standard error of the mean. FU, follow-up; IFN-γ, interferon γ; IL-2/4/10/17A, interleukin 2/4/10/17A; LVAD, left ventricular assist device; pre-LVAD, prior to LVAD implantation
